# A Microfabricated Branch Selection Platform for Quantitative Measurement of Leader–Follower Interaction Strength and Interaction Range in Collective Cell Migration

**DOI:** 10.3390/mi17040449

**Published:** 2026-04-05

**Authors:** Taichi Ashizawa, Kei Yamamoto, Kazuhiro Tsuneishi, Kenji Yasuda

**Affiliations:** 1 Department of Physics, School of Advanced Science and Engineering, Waseda University, 3-4-1 Okubo, Shinjuku, Tokyo 169-8555, Japan; a4-taichi@toki.waseda.jp (T.A.); kei.y@ruri.waseda.jp (K.Y.); kazu140613@akane.waseda.jp (K.T.); 2 Department of Pure and Applied Physics, Graduate School of Advanced Science and Engineering, Waseda University, 3-4-1 Okubo, Shinjuku, Tokyo 169-8555, Japan

**Keywords:** collective cell migration, leader–follower interaction, microfabricated platform, branch selection assay, single-cell train migration, cell–cell interaction quantification, run length statistics, microfluidic cell migration assay

## Abstract

Collective cell migration plays essential roles in morphogenesis, wound healing, angiogenesis, and cancer invasion, yet quantitative measurement of leader–follower interaction strength and range remains challenging due to the lack of direct and scalable methods. Here, we present a microfabricated branch selection platform combined with a probabilistic analysis framework to quantitatively measure intercellular coupling in migrating single-cell trains. Cells migrate through microchannels with a width of one cell and encounter symmetric T-junctions at which each follower cell selects either the same branch as the preceding cell or the opposite branch. We show that branch selection sequences are captured by a first-order Markov process, with the resulting run length (cluster size) statistics following a geometric form determined by an interaction-dependent transition probability. This relationship enables direct estimation of an effective interaction parameter without requiring force measurements or molecular labeling. Monte Carlo simulations confirm that interaction strength is primarily encoded in run length statistics rather than overall left/right occupancy in symmetric junctions. Experiments with epithelial MDCK cells and endothelial MS-1 cells reveal distinct interaction signatures: MS-1 cells show significant repulsive coupling, whereas MDCK cells exhibit at most a weak attractive tendency at the leader-first follower interface, while rear clusters display repulsive signatures. Cluster order-resolved analysis further indicates that interaction effects are spatially localized near the front and do not propagate as sustained attraction along the train. These results establish the proposed platform as a scalable method for quantitative measurement of interaction strength and interaction localization in collective cell migration.

## 1. Introduction

Collective cell migration is a fundamental biological process underpinning embryonic morphogenesis, wound healing, angiogenesis, and cancer invasion [1,2,3,4,5]. In many contexts, cells migrate as coordinated groups, with the motion of follower cells being biased through short-range mechanical and biochemical coupling [3,4]. Quantitatively characterizing the strength and spatial range of this leader–follower coupling is essential for understanding how directional information propagates within migrating collectives. A central concept in collective migration is functional heterogeneity along the migration axis. Cells at the advancing front often display dominant protrusive activity and polarity, and are frequently referred to as leader cells [3,5,6,7,8,9,10]. Such leader-like behavior has been linked to intracellular signaling dynamics (e.g., Rac/RhoA-related pathways) and the ability to sense and integrate external cues. In vascular morphogenesis, for example, tip cells guide stalk cells during sprouting angiogenesis [11,12]. Importantly, however, “leader” and “follower” are not always fixed identities; depending on the biological context, leader roles can be stable over long timescales (e.g., tracheal branch morphogenesis) or can be exchanged dynamically (e.g., border cell migration), indicating that leader–follower organization emerges from local interactions rather than being a purely cell-intrinsic state [2,12,13,14,15,16,17,18,19,20,21].

Accumulating evidence further indicates that leader emergence and maintenance are regulated by localized mechanical interactions within the collective. Mechanical interactions among neighboring cells can suppress leader-like fates in adjacent cells even before leader cells fully differentiate [22]. Moreover, recent optogenetic induction of leader-like phenotypes suggests that leader influence may be highly localized, primarily affecting the immediate neighboring follower cell [23]. In turn, rear cells contribute to collective coordination by maintaining cohesion and transmitting forces and signals across the group [24]. Together, these observations motivate the need for measurement methods that can directly quantify not only whether leader–follower coupling exists but also how far such influence extends along the migration axis. Recent studies and reviews have further emphasized the dynamic nature of leader–follower organization and the role of confinement in shaping collective migration phenotypes, motivating quantitative geometry-controlled assays for interaction inference [21,25,26,27,28].

The collective behavior of migrating cells is shaped not only by biochemical cues (e.g., VEGF in angiogenesis) but also by the geometry and confinement of the surrounding microenvironment [29,30]. In practice, confinement and microenvironmental geometry provide a powerful route for isolating and quantifying interaction rules [31,32,33,34,35,36]. In vitro studies using microfluidic confinement have shown that narrow channels can promote alignment and cohesive motion, whereas wider channels may reduce coordination by increasing degrees of freedom [37,38]. These findings are consistent with in vivo observations in developing embryos, where the migration efficiency of neural crest cell clusters depends on an optimal confinement width [39]. Similarly, micropatterned substrates can regulate cytoskeletal organization and protrusion dynamics through geometry-dependent constraints [40]. Beyond cell migration assays, agarose has been widely used in microfluidic systems and biodevices, including pathogen detection, droplet-based PCR, and droplet-formation physics [41,42,43], supporting its versatility as a microdevice material. Branching microenvironments further provide discrete decision points at which intercellular influence can be read out as path-selection statistics [44,45]. While these studies establish that confinement modulates collective dynamics, many analyses focus on leader behavior or bulk migration descriptors rather than providing a direct quantitative readout of local leader–follower coupling. A recent micropatterning study highlighted that contact-dependent behaviors such as contact inhibition of locomotion (CIL) and contact following locomotion (CFL) depend on the geometry and location of cell–cell contact sites [46], underscoring the need for assays that quantify local interaction rules under controlled spatial constraints.

Recently, we introduced an agarose-based microfabricated branching assay in which follower cells at a symmetric bifurcation frequently select the opposite branch from their predecessors, deviating from the independent Bernoulli expectation [47]. Here, we extend that feasibility demonstration into a quantitative platform by formulating sequential branch selection as a first-order Markov process and show that run length (cluster size) statistics follow a geometric form determined by an interaction-dependent transition probability. This formulation enables direct estimation of an effective interaction parameter and its position dependence along a train without force measurements or molecular labeling. We validate the framework through Monte Carlo simulations and experiments with epithelial MDCK cells and endothelial MS-1 cells, demonstrating distinct position-dependent interaction signatures. This platform provides a scalable and generalizable measurement tool for quantitative comparison of intercellular coupling across cell types and microenvironmental conditions.

## 2. Materials and Methods

### 2.1. Cells

We utilized a mouse-derived endothelial-like cell line, MILE SVEN 1 (MS-1; CRL-2279, ATCC, Manassas, VA, USA), and a canine-derived epithelial-like cell line, Madin-Darby canine kidney (MDCK) cells (RCB0995, RIKEN BioResource Research Center, Ibaraki, Japan). These cell lines were maintained at 37 °C in a humidified incubator with 5% CO_2_, using Dulbecco’s modified Eagle’s medium (DMEM; Gibco, Thermo Fisher Scientific, Waltham, MA, USA) supplemented with 10% heat-inactivated fetal bovine serum (FBS; Gibco, Waltham, MA, USA) and 100 U/mL penicillin-streptomycin (Gibco) as the culture medium for MS-1 cells and minimum essential medium Eagle (MEM; N4655, Merck KGaA, Darmstadt, Germany) with 10% FBS, 0.1 mM non-essential amino acids (NEAA; 11140-050, Gibco), and 100 U/mL penicillin-streptomycin as the culture medium for MDCK cells.

### 2.2. 1064/1480-nm Laser Photothermal Microfabrication System

A laser photothermal microfabrication system operating at 1064 nm and 1480 nm was used to create agarose microstructures, following the methods detailed in previous reports [38,47,48]. Specifically, the system integrates a laser-emitting module (PYL-1-1064-M, IPG Laser GmbH, Burbach, Germany; RLM-1-1480, IPG Laser, Oxford, MA, USA) with an inverted brightfield microscope (IX70, 20× objective, PLAN Apo, Olympus, Tokyo, Japan) for precise laser microfabrication. In this system, the laser beams for spot heating are controlled by a shutter controller (FSH-C, SIGMA software interface. To aid in the microfabrication process, the agarose-coated culture dish is mounted on a motorized X-Y microscope stage (SIGMA KOKI Co., Ltd., Tokyo, Japan), which is managed by a feedback stage controller (FC-101B, SIGMA KOKI Co., Ltd., Tokyo, Japan). Additionally, the stage position can be fine-tuned manually using a joystick controller (FJ-401B, SIGMA KOKI, Tokyo, Japan), allowing for adaptable and accurate adjustments throughout the fabrication process.

### 2.3. Agarose Microstructure Fabrication on Cultivation Dish

A 35 mm tissue culture dish (AGC TECHNO GLASS, Shizuoka, Japan) was initially treated with a plasma etching device (PIB-20, Vacuum Device, Ibaraki, Japan) for 5 min at a discharge current of 30 mA under 13.3 Pa pressure, enhancing its hydrophilicity. Then, a thin and uniform agarose gel layer was formed on the dish by spinning 3.0 wt% sol-state agarose droplets (low melting, analytical grade; Promega, Madison, WI, USA) using a spin coater (IH-D7, MIKASA, Tokyo, Japan) at 500 rpm for 3 s, followed by 3000 rpm for 18 s. During agarose microfabrication, the culture medium in the dish absorbs laser energy at the irradiated focal point, locally heating and dissolving the thin agarose gel layer at the laser spot. By adjusting the laser intensity, the dimensions of the adhesive regions can be precisely controlled, enabling tailored microfabrication designs even during cultivation.

Microchannels were fabricated in agarose gel using a photothermal etching technique, as previously described [47]. Briefly, a thin agarose gel layer was prepared on a glass-bottom dish by spin-coating 3.0 wt% sol-state low-melting agarose, as described above. Localized heating was generated with a focused infrared laser (1480 nm) to induce controlled melting of the agarose.

### 2.4. Cell Cultivation in Agarose Microstructures

A chamber (0.5 mm wide and 2 mm long) was fabricated as an agarose-free region within a thin agarose layer on a culture dish using the photothermal microfabrication technique. Then, 3 × 10^4^ cells were seeded onto the agarose-free reservoir region of the culture dish and incubated for 1 h at 37 °C in a humidified atmosphere containing 5% CO_2_. As cells in the agarose-free reservoir adhered to the dish, the unattached cells over the agarose-coated regions were removed during the medium exchange. When cells in the reservoir chamber reached near-confluence, microchannels were added to the adjacent agarose layer using a photothermal microfabrication system. Single-cell-wide linear microchannels (width: 10– 15 μm) were fabricated, terminating in symmetric T-shaped branching junctions. The branching geometry was designed to ensure geometric symmetry between the left and right branches, minimizing intrinsic directional bias. Following cell entry into the microchannels, a series of time-lapse imaging sessions was initiated to monitor and record the collective migration dynamics of cells.

### 2.5. Time-Lapse Recording and Analysis of Cellular Dynamics

Time-lapse images of cells within topographical constraints were captured at 10-min intervals using two systems. The first was an agarose microfabrication system equipped with a stage-top microscope incubation chamber (TOKAI HIT, Shizuoka, Japan) and a motorized X-Y microscope stage for software-controlled positioning. This system used a digital camera (1501M-GE-TE, Thorlabs Inc., Newton, NJ, USA) for imaging. The second system was an automated image recording setup called CytoWatcher (WSL-1800, Atto Ltd., Tokyo, Japan). During the time-lapse observation, the temperature was maintained at 37 °C and the pH was maintained at the incubator’s growth medium pH. To regulate the pH, a gas mixer (Kofloc Kojima Instruments Inc., Tokyo, Japan) was employed to combine air with CO_2_ gas. This gas mixture was filtered and introduced into the water bath of the stage-top microscope incubation system through a tube.

### 2.6. Branch Selection Measurement Assay and Data Analysis

Branch selection experiments were performed using symmetric T-shaped bifurcation patterns designed to minimize geometric bias. The experimental workflow followed the branching assay introduced in our previous study [47], with additional analyses for quantitative comparison with probabilistic models.

#### 2.6.1. Branch Selection Sequence Extraction

Time-lapse brightfield images were used to determine the migration direction each cell chose at the bifurcation. For each migrating train, the branch choice of the first (front) cell was used as the reference direction. Each subsequent (rear) cell was classified as a same-branch event (choosing the same branch as the former cell) or an opposite-branch event (choosing the opposite branch).

#### 2.6.2. Cluster Size (Run Length) Definition

A cluster was defined as a contiguous sequence of cells that chose the same branch direction. The cluster size *n* denotes the number of consecutive cells in a cluster (number of cells in series). Cluster-size distributions were computed separately for the first cluster (front-cell cluster) and for the second and subsequent clusters (second and subsequent n-th rear-cell clusters).

#### 2.6.3. Null Model Comparison

As a null model, we assumed independent branch selection with probability *p* for the same-branch event (Bernoulli trials). For symmetric bifurcations, *p* is expected to be approximately 0.5. Under this assumption, the run length distribution follows a geometric distribution, which provides a baseline for detecting interaction-driven deviations.

#### 2.6.4. Interaction Parameter Estimation

To quantify effective leader–follower or front–rear interactions of cells, we used the interaction-augmented probabilistic framework described in Section 3.2. The effective interaction strength was estimated from the observed cluster size (number of cells in series) distributions by comparing the measured mean run length with the null model prediction.

#### 2.6.5. Geometrical Baseline Calibration

Although the T-junctions were designed to be symmetric, small fabrication errors could in principle introduce a baseline bias in the branching probability *p*. In the present study, direct calibration using repeated measurements in the same microchannel was not feasible because each microchannel was used only once for the migration experiment. Therefore, we evaluated the baseline symmetry using two complementary approaches.

First, the geometric symmetry of the fabricated T-junctions was assessed using the fabrication precision enabled by automated agarose photothermal microfabrication, confirming that the left and right branches were formed with comparable widths and lengths within the resolution of the fabrication process.

Second, to experimentally validate the absence of directional bias, we analyzed the branch choice outcomes of front (leader) cells across multiple independently fabricated symmetric T-junctions prepared in the same dish under identical conditions. Because the front cell is the first to encounter the junction, its left/right choice provides a direct readout of baseline geometric bias. Across these junctions, the front cell selected the left and right branches with approximately equal frequencies, indicating negligible directional bias and supporting the approximation p≈0.5.

Accordingly, we used p=0.5 as the baseline probability for the main analyses unless otherwise stated.

We note that the estimated interaction parameters primarily rely on run length statistics (correlations), which are less sensitive to small residual baseline bias than marginal left/right occupancy statistics.

### 2.7. Monte Carlo Simulations

Monte Carlo simulations were performed to validate theoretical predictions. Sequential branch selection sequences were generated using transition probabilities defined by specified interaction parameters (α=−0.2,0,0.2). Each simulation consisted of 20 independent trials of 20-cell sequences. Run-length distributions were computed and compared with theoretical predictions.

### 2.8. Statistical Analysis

All analyses were performed using standard statistical procedures based on the equations described in Section 3.2. Specifically, the interaction parameter was estimated from run length statistics using the mean-based estimator and/or maximum likelihood estimation under the geometric model. Confidence intervals were obtained by nonparametric bootstrap resampling (10,000 iterations). All calculations were reproduced by independent re-analysis from the exported run length tables. Likelihood ratio tests (LRT) against the independence null model (α=0; $P_{jj}=0.5$) were evaluated using a parametric bootstrap procedure: synthetic run length samples were generated under the null model with the same number of clusters *M* as in the experimental dataset, the LRT statistic Λ was computed for each replicate, and *p*-values were obtained from the empirical null distribution.

## 3. Results and Discussion

Building on our previous study that introduced the T-junction branch-selection assay (Figure 2 in [47]), we present a more detailed quantitative characterization of the same measurement concept. Specifically, we formalize the probabilistic framework, define interaction-sensitive statistics, and validate the platform using two distinct cell types (MS-1 as an endothelial cell model and MDCK as an epithelial cell model) in symmetric T-junction patterns.

### 3.1. Conceptual Idea of Front–Rear Cell Interaction Measurement in Migrating Single-Cell Trains Using Path Selection Frequencies of Branched Micro-Pathways

**Figure 1:**
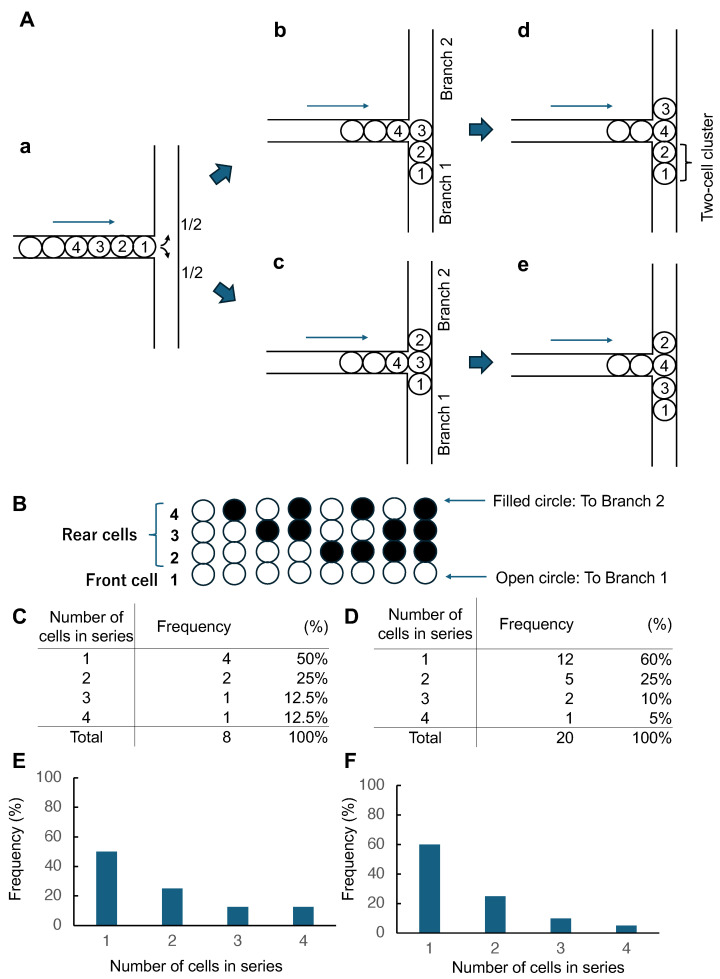
Principle of the branch selection assay and definition of run length (cluster size). (**A**). In a symmetric T-junction, the front cell reaches the bifurcation (**a**) and each follower cell subsequently selects either the same branch as the preceding cell (“same-branch”) (**b**,**d**) or the opposite branch (“opposite-branch”) (**c**,**e**). Arrows indicate the migration direction, and numbers label the order of cells within the single-cell train (1 = front/leader cell). (**B**). Example enumeration for a four-cell train under the independence (Bernoulli) null model (p=0.5), where open/filled circles indicate same/opposite branch relative to the front-cell branch. (**C**). Distribution of the front-cluster size (consecutive same-branch choices starting from the front cell). (**D**). Distribution of run length (cluster size) across the entire sequence. (**E**,**F**). Graphical representation of (**C**,**D**).

Figure 1 summarizes the branch selection assay used to quantify leader–follower coupling in confined single-cell trains. Cells migrate in microchannels with a width of one cell and encounter a symmetric T-junction; the first (leader) cell selects one branch, and each subsequent follower cell selects either the same branch (same-branch) or the opposite branch (opposite-branch). This converts continuous intercellular influence into a binary sequence of branch choices.

As a null model, we assume independent Bernoulli branching with probability *p* of selecting either branch (for an ideal symmetric junction, p=0.5). Under this independence assumption, the expected cluster size (run length) distributions can be calculated for a given train length (Figure 1B–F). Deviations from the null model distributions indicate effective interactions: attraction increases the frequency of long runs (large clusters), whereas repulsion enriches single-cell runs. In the following, we quantify these deviations using a probabilistic framework that links run length statistics to an interaction-dependent transition probability.

### 3.2. Probabilistic Analysis of Branch Selection in Confined Micro-Pathways

In order to link branch selection statistics to an interaction parameter, we model sequential choices at a symmetric T-junction as a first-order Markov process. Let $X_{i}\in \left\{L,R\right\}$ denote the branch selected by the *i*-th cell and define the transition probability matrix

(1) $$P_{jk}=\mathrm{Pr}\left(X_{i+1}=k|X_{i}=j\right),$$

(2) $$P_{jj}=p+\alpha ,$$

(3) −p≤α≤1−p.

(4) $$P\left(n\right)={\left(P_{jj}\right)}^{n-1}\left(1-P_{jj}\right),$$

(5) $$\langle n\rangle =\frac{1}{1-P_{jj}},$$

where j,k∈{L,R}. We focus on the same-branch transition probability $P_{jj}$ and write $P_{jj}=p+\alpha$, where *p* is the baseline probability in the absence of interactions (for an ideal symmetric junction, p=0.5) and α is an effective interaction parameter (positive: attraction; negative: repulsion). The run length distribution follows a geometric form and the expected run length is $\langle n\rangle =1/\left(1-P_{jj}\right)$, enabling direct estimation of $P_{jj}$ (and thus α) from observed cluster sizes.

**Figure 2:**
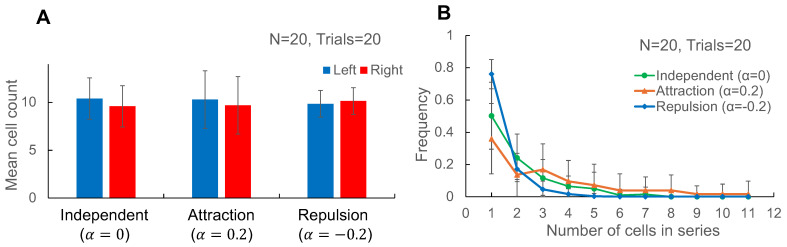
Probabilistic analysis of branch selection in confined micro-pathways. (**A**). Mean (±SD) numbers of cells that entered the Left branch (the branch selected by the front cell) and Right branch (the opposite branch) for trains of N=20 cells. Bars summarize 20 independent simulation trials for each interaction condition: independent branching (α=0), attractive coupling (α = 0.2), and repulsive coupling (α=−0.2). (**B**). Corresponding run length (cluster size) distributions computed from the same simulated sequences (Mean ± SD of 20 independent simulation trials). Attractive coupling increases the occurrence of large clusters, whereas repulsive coupling enriches single-cell clusters.

Monte Carlo simulations (Figure 2) confirm that interaction strength is best reported by run length statistics (correlations) rather than by marginal left/right occupancy in symmetric junctions.

#### Estimation of Interaction Parameter from Run Length Statistics

As shown in the preceding analysis, the run length distribution follows a geometric distribution (Equation (4)) determined by the same-branch transition probability $P_{jj}$, and its mean run length is given by Equation (5). This relationship provides a direct method for estimating the interaction parameter from experimentally observed branch-selection sequences.

Let ${\langle n\rangle }_{\mathrm{obs}}$ denote the experimentally measured mean run length. From Equation (5), the same-branch transition probability can be estimated as

(6) $${\hat{P}}_{jj}=1-\frac{1}{{\langle n\rangle }_{\mathrm{obs}}}.$$

Using the relation $P_{jj}=p+\alpha$, the interaction parameter is estimated as

(7) $$\hat{\alpha }={\hat{P}}_{jj}-p.$$

As described in Section 2.6.5, baseline calibration confirmed no measurable directional bias (p≈0.5) under our experimental conditions; therefore, for the symmetric junction we use p=0.5 in the simplified estimator below.

Because geometrical baseline calibration confirmed negligible directional bias in the present symmetric junctions (Section 2.6.5), we use p=0.5 in the following simplified estimator.

For an ideal symmetric junction (p=0.5), this simplifies to

(8) $$\hat{\alpha }=\frac{1}{2}-\frac{1}{{\langle n\rangle }_{\mathrm{obs}}}.$$

This estimator provides a direct physical interpretation: positive values of $\hat{\alpha }$ indicate attractive leader–follower coupling, whereas negative values indicate repulsive interactions. When $\hat{\alpha }=0$, branch selection is consistent with independent Bernoulli trials.

Alternatively, the interaction parameter can be estimated using maximum likelihood estimation (MLE). Let $\left\{n_{1},n_{2},\ldots ,n_{M}\right\}$ denote the set of observed run lengths extracted from branch selection sequences, where *M* is the total number of observed run length events (clusters).

Under the geometric distribution model (Equation (4)), the likelihood function is

(9) $$L\left(P_{jj}\right)=\prod_{i=1}^{M}{\left(P_{jj}\right)}^{n_{i}-1}\left(1-P_{jj}\right),$$

and maximization of this likelihood yields

(10) $${\hat{P}}_{jj}=\frac{\sum_{i=1}^{M}\left(n_{i}-1\right)}{\sum_{i=1}^{M}n_{i}}.$$

This estimator is equivalent to the mean-based estimator and provides a statistically optimal estimate under the geometric model assumption.

Thus, run length statistics obtained from branch selection experiments provide a direct and quantitative estimator of the effective front–rear (leader–follower) interaction strength without requiring force measurements, molecular labeling, or high-resolution force inference.

Therefore, this formulation enables direct experimental quantification of intercellular interaction strength from observed branch selection sequences.

### 3.3. Measurement of Leader–Follower Interaction Strength Using Symmetric T-Junction Branching Experiments

**Figure 3:**
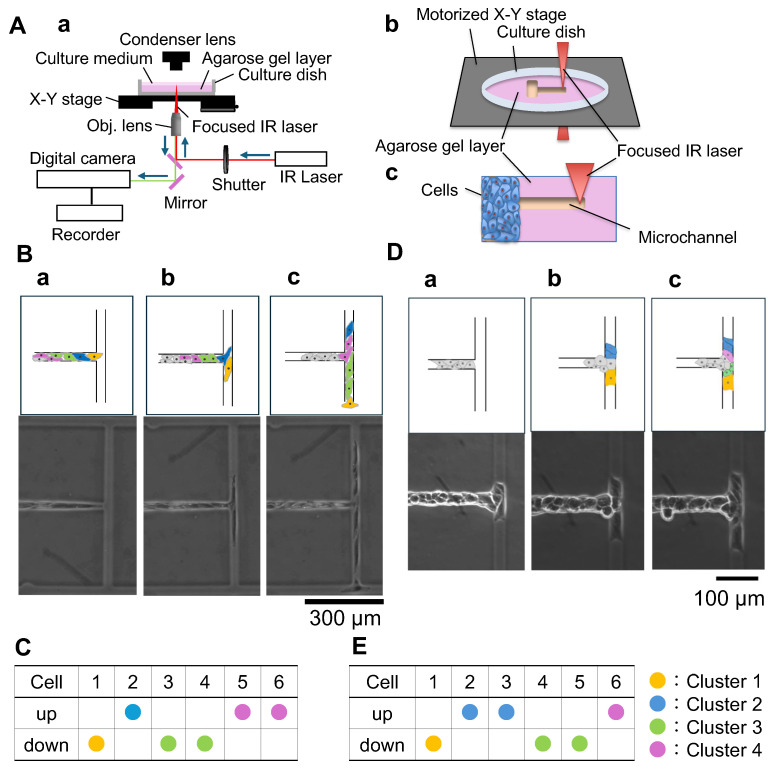
Symmetric T-junction branching experiments for measuring leader–follower interactions. (**A**) Experimental system: (**a**) photothermal microfabrication system using 1064/1480 nm laser irradiation (Red and blue lines indicate the optical paths: the red line shows the infrared (IR) laser pathway and the blue line shows the microscope imaging light path; arrows indicate the propagation directions of each beam); (**b**) in situ fabrication of agarose microchannels during cell cultivation; (**c**) schematic of the agarose photothermal microfabrication procedure used to carve microgroove pathways during cell culture. (**B**) Representative migration sequence of endothelial MS-1 cells in a symmetric T-junction; colored overlays indicate the cluster order (yellow: 1st, blue: 2nd, green: 3rd, purple: 4th), using the same color scheme throughout this figure; (**a**) front cell reaches the junction (cluster 1, yellow); (**b**) second cell selects the opposite branch (cluster 2, blue); (**c**) third cell cluster selects the same branch as the front cell cluster 3, green), followed by a cluster selecting the opposite branch (cluster 4, purple). (**C**) Example branch selection sequence of MS-1 cells; numbers indicate the cell order in the train. ‘Up/Down’ denotes the selected branch at the junction. Colored circles (yellow, blue, green, and purple) indicate the cluster order (1st–4th clusters), consistent with the color coding used in (**B**). (**D**) Representative migration sequence of epithelial MDCK cells; colored overlays indicate the cluster order (yellow: 1st, blue: 2nd, green: 3rd, purple: 4th), using the same color scheme as in (**B**) and panels (**C**,**E**). (**a**) the front (leader) cell reaches the junction (cluster 1, yellow); (**b**) the next cluster selects the opposite branch (cluster 2, blue); (**c**) the subsequent cluster selects the same branch as the front cell (cluster 3, green), followed by a cluster selecting the opposite branch (cluster 4, purple). (**E**) Example branch selection sequence of MDCK cells. Numbers indicate the cell order in the train. ‘Up/Down’ denotes the selected branch at the junction. Colored circles (yellow, blue, green, and purple) indicate the cluster order (1st–4th clusters), consistent with the color coding used in (**D**).

We experimentally validated the assay using agarose microfabricated symmetric T-junctions (Figure 3). Branch selection sequences showed deviations from the independence model that were dependent on cell-type, motivating the quantitative parameter estimation in the next subsection.

### 3.4. Quantitative Estimation of Interaction Strength and Interaction Range from Experimental Cluster Size Distributions

**Figure 4:**
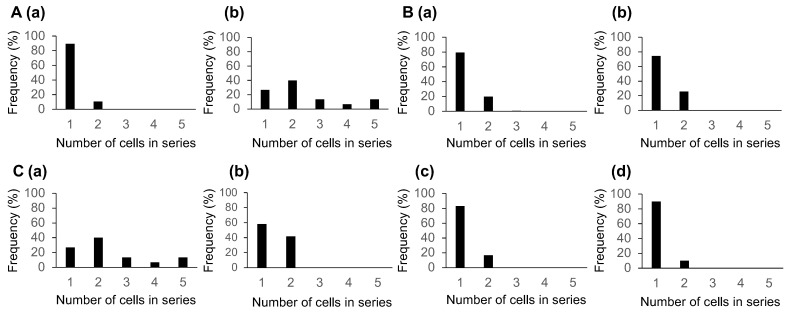
Cluster size distributions and position-dependent interaction signatures in symmetric T-junction experiments. (**A**) Front-cell cluster size distributions: (**a**) MS-1 endothelial cells (N = 19 trains) and (**b**) MDCK epithelial cells (N = 15 trains). (**B**) Rear-cell cluster size distributions pooled across all subsequent clusters: (**a**) MS-1 cells (N = 19 trains, n = 132 clusters) and (**b**) MDCK cells (N = 15 trains, n = 39 clusters). (**C**) Cluster order-resolved distributions for MDCK cells (N = 15 trains): (**a**) first cluster (front-cell cluster, same as (**A**(**b**))); (**b**) second cluster; (**c**) third cluster; (**d**) fourth cluster. The interaction signatures change with cluster order, indicating that leader influence is localized near the front and does not propagate monotonically along the train.

To quantitatively determine leader–follower interaction strength, cluster size distributions obtained from symmetric T-junction experiments (Figure 4) were analyzed using the probabilistic framework described in Section 3.2 (Table 1).

The interaction parameter α was estimated from the mean cluster size using Equation (8). Statistical uncertainty was quantified using bootstrap resampling (10,000 iterations).

For MS-1 endothelial cells, the front-cluster interaction parameter was estimated as

$${\hat{\alpha }}_{1}=-0.405\;\left(95\%\;CI:\;-0.50\;\mathrm{to}\;-0.29\right),$$

indicating a significant repulsive interaction between the leader and follower cells. Rear clusters showed reduced but still negative interaction strength:

$${\hat{\alpha }}_{rear}=-0.313\;\left(95\%\;CI:\;-0.37\;\mathrm{to}\;-0.26\right),$$

indicating persistent repulsive coupling.

For MDCK epithelial cells, the front-cluster interaction parameter was

$${\hat{\alpha }}_{1}=+0.083\;\left(95\%\;CI:\;-0.056\;\mathrm{to}\;+0.174\right),$$

which is consistent with a weak attractive tendency but is not statistically distinguishable from the independence model (α=0) based on the confidence interval alone.

However, cluster order-resolved analysis revealed a rapid decay of interaction strength. The second cluster showed

$${\hat{\alpha }}_{2}=-0.206\;\left(95\%\;CI:\;-0.36\;\mathrm{to}\;-0.10\right),$$

while the third and fourth clusters exhibited stronger repulsion:

$${\hat{\alpha }}_{3}=-0.357,\;{\hat{\alpha }}_{4}=-0.409.$$

These results demonstrate that when, attractive leader–follower interactions are present, they are confined to the interface between the leader and first follower, and do not propagate further along the cell train.

Here, we define the effective interaction range as the farthest position (cluster order) at which the estimated interaction parameter α remains distinguishable from the independence null model (α=0) based on the bootstrap confidence interval and/or the likelihood ratio test. Under this definition, interaction effects are effectively limited to approximately one cell length in the present MDCK trains: the front cluster shows at most a weak attractive tendency, whereas rear clusters approach the null model or exhibit weakly repulsive signatures.

These results establish that the symmetric T-junction platform enables quantitative measurement of both interaction strength and interaction range in collective cell migration.

These results also demonstrate that leader–follower interaction is spatially limited, with an effective interaction range of approximately one cell length. This finding provides a direct experimental framework for quantitative measurement of interaction range in collective cell migration using microfabricated branching assays.

#### Statistical Validation Using Likelihood Ratio Test

To statistically validate deviations from the independence model (α=0), we compared the likelihood of observed run length distributions under the interaction model (α≠0) and the null model (α=0). Because some datasets contain a limited number of clusters, *p*-values were evaluated using a parametric bootstrap likelihood ratio test under the geometric null model ($P_{jj}=0.5$).

MS-1 clusters showed significant deviation from the null model (front cluster: $p=8\times 10^{-5}$; rear clusters: $p<10^{-5}$), confirming strong repulsive interaction signatures. For MDCK cells, the front-cluster distribution did not significantly deviate from the null model (p=0.37), indicating at most a weak attractive tendency. In contrast, rear clusters significantly deviated from the null model ($p=8\times 10^{-5}$) and cluster order analysis showed significant repulsive signatures in the third and fourth clusters (p<0.01), whereas the second cluster was not distinguishable from the null model (p=0.11).

### 3.5. Methodological Implications

This work establishes a branch selection platform as a quantitative measurement method for leader–follower coupling in collective cell migration. By converting continuous intercellular interactions into discrete branch choice sequences at a symmetric bifurcation, the method enables direct estimation of an effective interaction parameter and its position dependence along a single-cell train using a minimal first-order Markov model. A key practical advantage is that interaction strength and localization can be extracted from time-lapse trajectories and run length statistics without requiring traction-force microscopy, force sensors, or molecular labeling, thereby facilitating systematic comparison across cell types and microenvironmental conditions.

Beyond symmetric T-junctions, the same probabilistic framework can be generalized to (i) junctions with controlled geometric bias (by calibrating the baseline probability), (ii) multi-way bifurcations, and (iii) networks with repeated decision points. These extensions make the platform a scalable tool for quantifying how directional information propagates (or fails to propagate) through confined migrating collectives as well as for benchmarking the effects of pharmacological or genetic perturbations on intercellular coupling in a device-independent manner.

Related work has shown that geometric confinement can both regulate and reveal collective-migration phenotypes, including leader-like behavior, in both experimental micropatterns [25,34,35,36] and theory models of confined collectives [49,50,51,52]. Our branch selection assay extends this line of research by operationalizing confined migration into a sequence of discrete junction decisions, enabling (i) direct estimation of an effective interaction strength parameter from run length statistics and (ii) cluster order-resolved evaluation of interaction localization (effective range) along the train. This strategy is experimentally practical and scalable; it relies only on standard time-lapse trajectories under well-defined geometry without requiring traction-force measurements, molecular labeling, or invasive perturbations, thereby facilitating systematic comparisons across cell types, microenvironmental conditions, and perturbations.

### 3.6. Limitations

Nonetheless, several limitations should be noted, as summarized below.

#### 3.6.1. Influence of Cell Shape and Deformation

While the probabilistic estimator does not assume spherical cells (Figure 1 is a conceptual schematic), real cells deform and elongate under confinement. Such shape changes can alter the junction dwell time, cell–cell contact area, and transient branch occupancy (partial occlusion), potentially modulating the effective same-branch transition probability $P_{jj}$ and consequently the estimated interaction parameter α. In the present framework, these effects are absorbed into an effective interaction parameter that integrates mechanical/biochemical coupling and shape-mediated occupancy. Future work could stratify events by aspect ratio near the junction or incorporate automated shape tracking to quantify and control this source of variability.

#### 3.6.2. Model Order and Higher-Order Interactions

The present analysis assumes first-order (nearest-neighbor) dependence, i.e., each follower cell’s branch choice depends primarily on the immediately preceding cell. Although this is physically motivated by single-file confinement, it is possible that higher-order interactions, delayed responses, or collective memory effects may become relevant in other geometries or at higher densities. Such effects could be captured by higher-order Markov models or non-Markovian extensions.

#### 3.6.3. Stationarity Assumption and Position-Dependent Coupling

The geometric run length model assumes stationarity of the transition probability within each analyzed group. Our cluster order-resolved results indicate position-dependent effective coupling, suggesting that future work should explicitly model nonstationary transition probabilities along the train.

#### 3.6.4. Annotation Uncertainty and Run Length Extraction

Branch choice annotation and run length extraction introduce measurement uncertainty, particularly near the junction and at close cell spacing; automated tracking-based extraction and inter-annotator validation would improve throughput and reduce observer dependence.

#### 3.6.5. Effect of Limited Sample Size in Cluster Statistics

In Figure 4, the number of MDCK rear-cluster events (n=39) is relatively small, which can reduce statistical robustness by widening confidence intervals and increasing sensitivity to outliers or rare long runs. In the present study, we explicitly quantify this uncertainty using nonparametric bootstrap confidence intervals (10,000 resamples) and, where applicable, bootstrap-based likelihood ratio tests against the independence model. Nevertheless, increasing the number of independent trains/junctions and applying train-level resampling (clustered bootstrap) or hierarchical models would further strengthen robustness in future studies.

#### 3.6.6. Baseline Bias and Geometric Asymmetry

While baseline calibration indicated negligible directional bias in the present symmetric junctions, subtle geometric asymmetries or external cues may bias the baseline probability in other implementations, and should be calibrated when applying the method to new devices or cell types.

#### 3.6.7. Hydrodynamic Resistance Analogy and Junction Occupancy Effects

A potential physical contribution to the observed branch selection correlations is a transient change in the effective “resistance” of each branch when a cell enters the junction. In pressure-driven microfluidic networks, branching flows are often analyzed using an electrical circuit analogy in which pressure corresponds to voltage, flow rate to current, and hydrodynamic resistance to electrical resistance [44]. In our agarose microfabrication, the pathway is carved as a bottom groove and the top surface is open to the culture medium; therefore, a closed-channel pressure-driven flow is not assumed in the present experiments. Nevertheless, the circuit/resistance analogy provides a useful conceptual framework to describe how geometrical occupancy at a junction can bias downstream partitioning. By analogy, a cell partially occupying one branch near the bifurcation may transiently increase the effective resistance of that branch (e.g., through geometrical occlusion and shape-dependent confinement), biasing subsequent cells toward the alternative branch. Related junction phenomena have also been extensively studied in droplet splitting at T-junctions, where branch geometry and occupancy strongly influence downstream partitioning [45]. In the present framework, such geometry- and occupancy-mediated effects are not treated as separate variables but are naturally absorbed into the effective same-branch transition probability $P_{jj}$ and the inferred interaction parameter α. Future extensions could explicitly couple a resistance-based microfluidic network model with the probabilistic estimator to separate direct cell–cell coupling from occupancy-induced biases across different channel designs and cell types.

## 4. Conclusions

We have developed a microfabricated branch selection platform combined with a probabilistic analysis framework to quantitatively measure leader–follower interaction strength and interaction range in collective cell migration. By analyzing the run length distributions of branch selection sequences in branching microchannels with a width of one cell, we were able to directly estimate interaction parameters without requiring force measurements or molecular labeling. Experimental validation using MDCK and MS-1 cells revealed cell type-dependent interaction strengths and demonstrated that interaction effects decay with distance from the leader cell, indicating a finite interaction range primarily limited to adjacent cells. This platform provides a scalable and generalizable method for quantitative analysis of intercellular coupling and establishes a versatile experimental framework for investigating collective cell migration in controlled microenvironments.

## Figures and Tables

**Table 1:** Estimated interaction parameter α from cluster size statistics. Uncertainty was quantified using bootstrap resampling (10,000 iterations). Positive values indicate attractive leader–follower interactions, whereas negative values indicate repulsive interactions.

Dataset	Mean Cluster Size	$\hat{\alpha }$	SE	95% CI
MS-1 front cluster	1.105	−0.405	0.056	[−0.500, −0.292]
MS-1 rear clusters	1.230	−0.313	0.027	[−0.369, −0.264]
MDCK front cluster	2.400	+0.083	0.060	[−0.056, +0.174]
MDCK rear clusters	1.256	−0.296	0.044	[−0.386, −0.209]
MDCK cluster 2	1.417	−0.206	0.073	[−0.357, −0.100]
MDCK cluster 3	1.167	−0.357	0.078	[−0.500, −0.206]
MDCK cluster 4	1.100	−0.409	0.075	[−0.500, −0.269]

## Data Availability

The original contributions presented in this study are included in the article. Further inquiries can be directed to the corresponding author.
